# Supplementary material for: Bacterial Biofilm Formation Using PCL/Curcumin Electrospun Fibers and Its Potential Use for Biotechnological Applications
Source: Materials (Basel). 2020 Dec 6;13(23):5556. doi: 10.3390/ma13235556 (PMC7729789; doi:10.3390/ma13235556)
Supplement: Supplementary file 1 [file materials-13-05556-s001.pdf]

materials

Supplementary

Bacterial Biofilm Formation Using PCL/Curcumin Electrospun Fibers and Its Potential Use for Biotechnological Applications

[1] Daniella Alejandra Pompa-Monroy [1,2], Paulina Guadalupe Figueroa-Marchant [1], Syed G. Dastager [3,4], Meghana Namdeo Thorat [4], Ana Leticia Iglesias [1], Valentín Miranda-Soto [5], Graciela Lizeth Pérez-González [1,2] and Luis Jesús Villarreal-Gómez [1,2,*]

1 Facultad de Ciencias de la Ingeniería y Tecnología, Universidad Autónoma de Baja California, Blvd Universitario 1000, Unidad Valle de Las Palmas, 22260 Tijuana, Baja California, México; daniella.pompa@uabc.edu.mx (D.A.P.-M.);figueroa.paulina@uabc.edu.mx (P.G.F.-M.); aiglesias@uabc.edu.mx (A.L.I.); perez.graciela@uabc.edu.mx (G.L.P.-G.)
2 Facultad de Ciencias Químicas e Ingeniería, Universidad Autónoma de Baja California, Universidad #14418, Parque Internacional Industrial Tijuana, 22390 Tijuana, Baja California, México
3 Academy of Scientific and Innovative Research (AcSIR), AcSIR Headquarters CSIR-HRDC Campus, Postal Staff College Area, Sector 19, Kamla Nehru Nagar, 201002 Ghaziabad, India; sg.dastager@ncl.res.in
4 NCIM Resource Center, CSIR-National Chemical Laboratory, 411008, Pune, India; mn.thorat@ncl.res.in
5 Tecnológico Nacional de México/Instituto Tecnológico de Tijuana/Centro de Graduados e Investigación en Química, Calzada Del Tecnológico S/N, Fraccionamiento Tomas Aquino. 22414, Tijuana, Baja California, México; vmiranda@tectijuana.mx
* Correspondence: luis.villarreal@uabc.edu.mx

Received: 24 September 2020; Accepted: 27 October 2020; Published: date

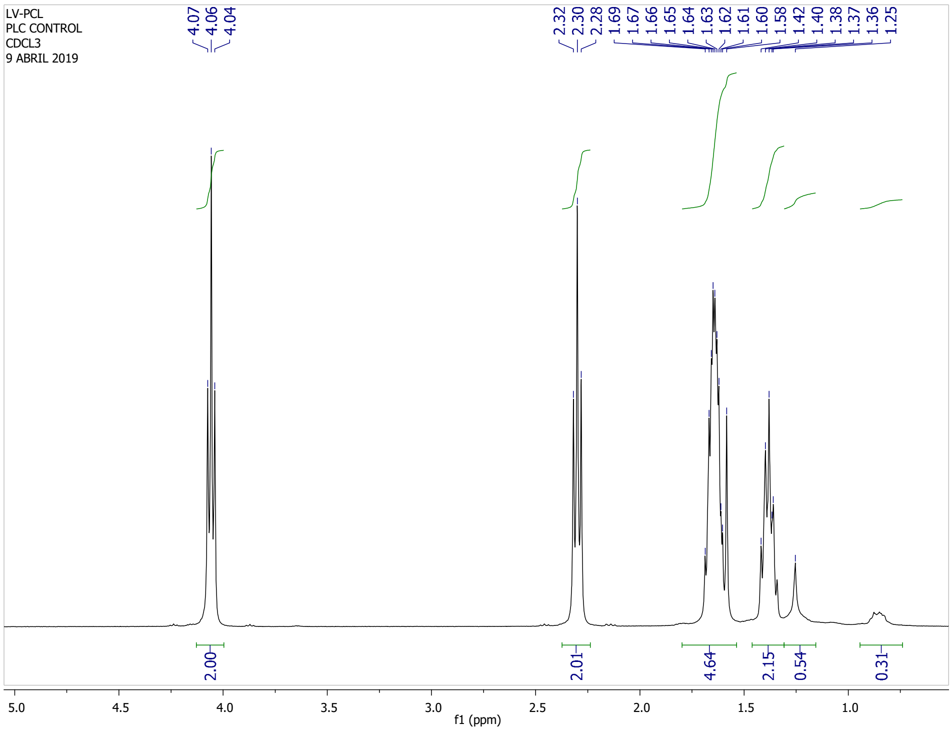

Figure S1. ^{1}H NMR of PCL control ($CDCl_3$).

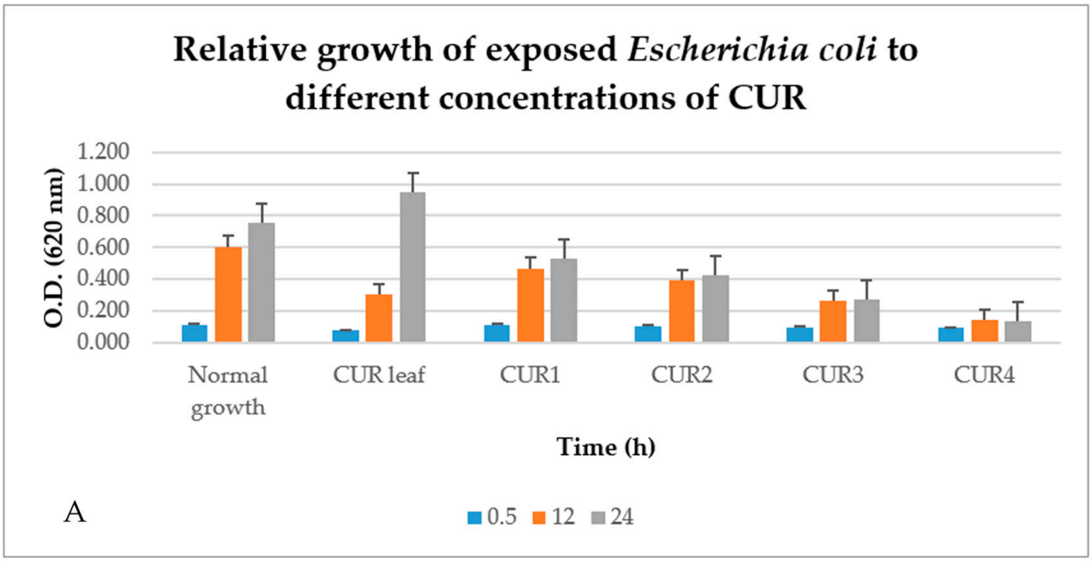

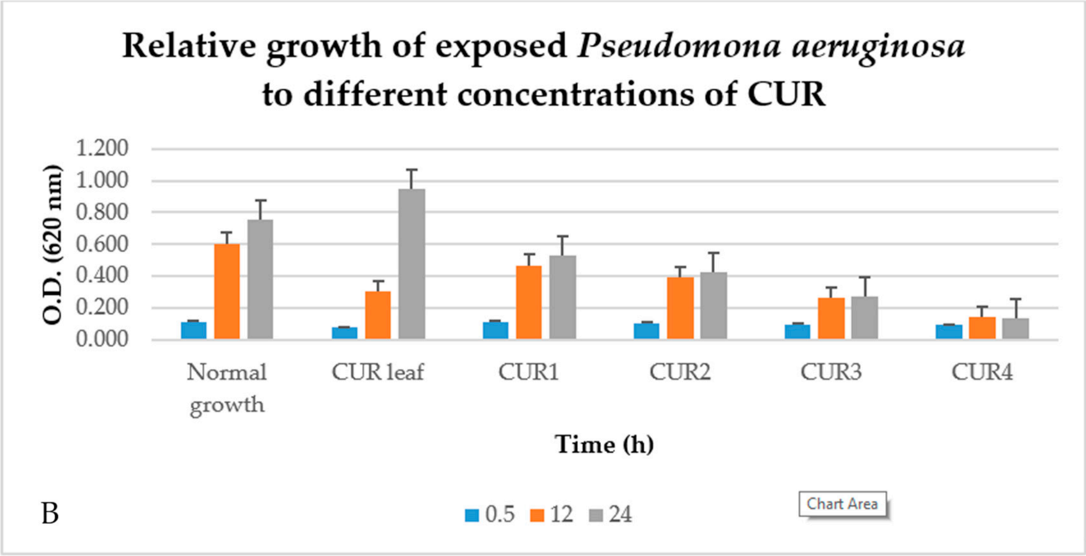

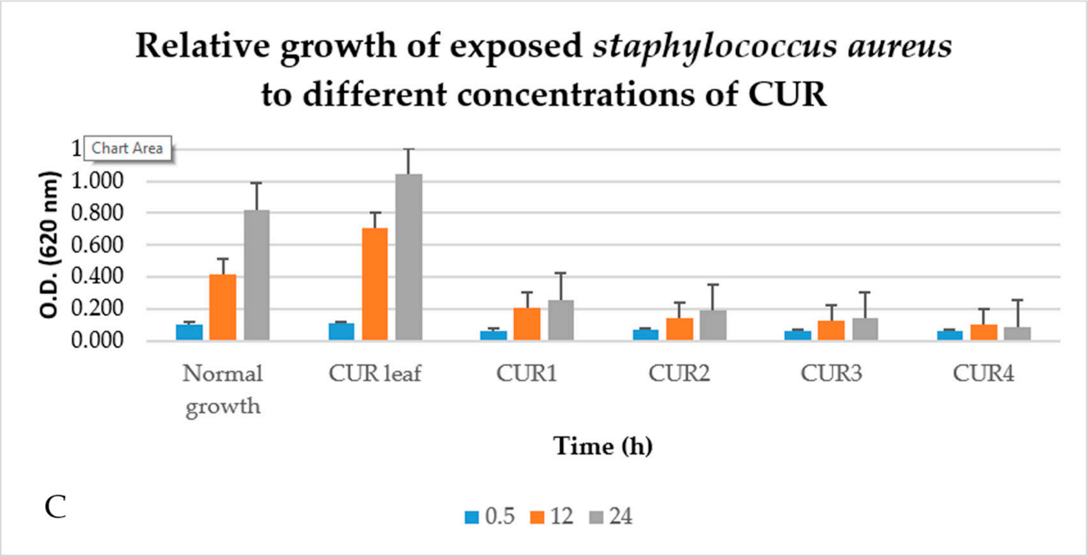

Figure S2. Relative growth of exposed bacteria to different concentrations of CUR. (**A**) *Escherichia coli*, (**B**) *Pseudomona aeruginosa*, (**C**) *Staphylococcus aureus*.

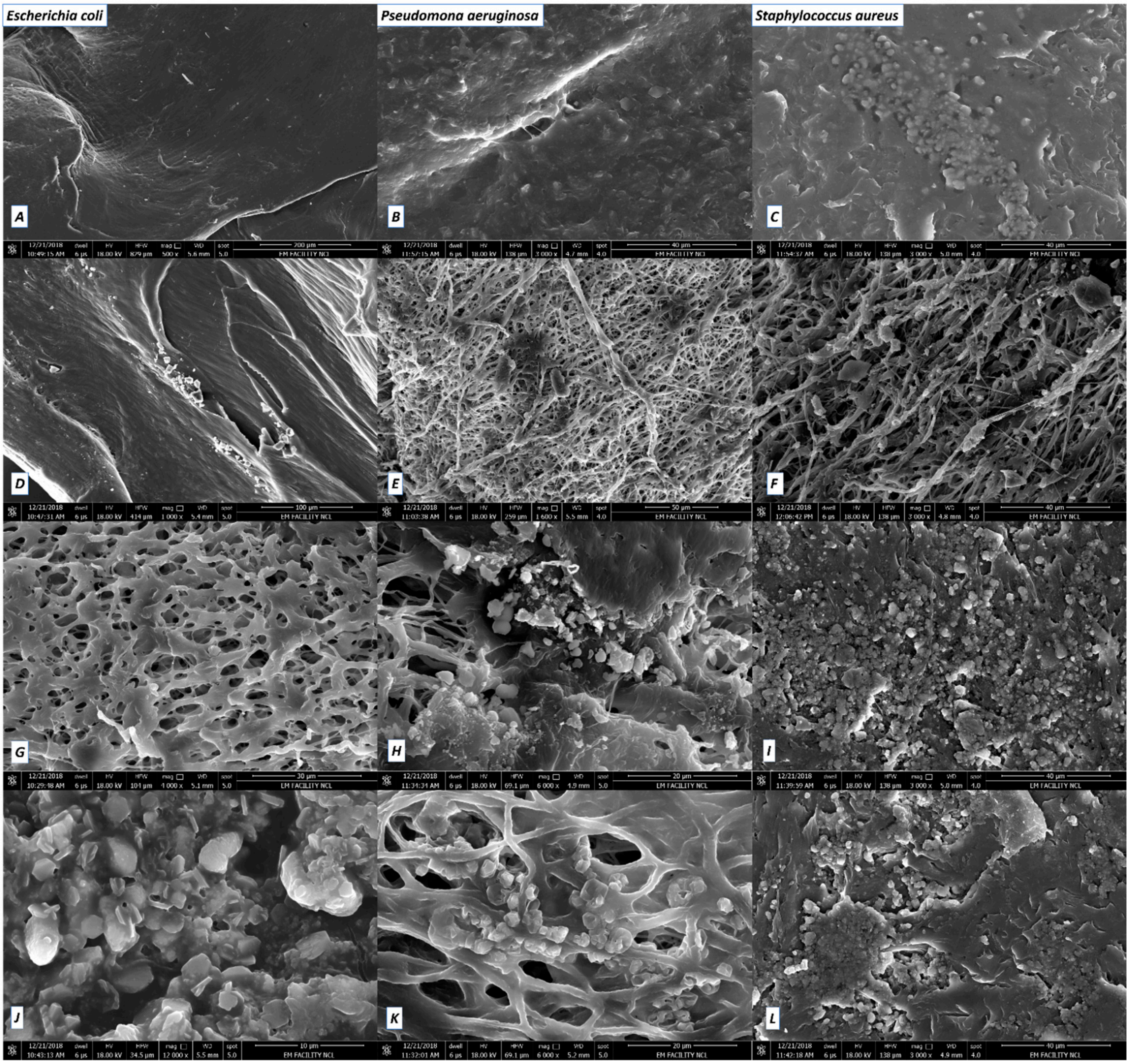

Figure S3. SEM images of exposed PCLc and PCL/CUR scaffolds with bacteria after 12 h of incubation. (**A**) *Escherichia coli* on PCLc fibers (500×), (**B**) *Pseudomona aeruginosa* on PCLc fibers (3000×), (**C**) *Staphylococcus aureus* on PCLc fibers (3000×), (**D**) *Escherichia coli* on PCL/CUR1 fibers (1000×), (**E**) *Pseudomona aeruginosa* on PCL/CUR1 fibers (1600×), (**F**) *Staphylococcus aureus* on PCL/CUR1 fibers (3,000×), (**G**) *Escherichia coli* on PCL/CUR2 fibers (4000×), (**H**) *Staphylococcus aureus* on PCL/CUR2 fibers at (6000×), (**I**) *Staphylococcus aureus* on PCL/CUR2 fibers (3000×), (**J**) *Escherichia coli* on PCL/CUR3 fibers (12000×), (**K**) *Staphylococcus aureus* on PCL/CUR3 fibers at (6000×), (**L**) *Staphylococcus aureus* on PCL/CUR3 fibers (3000×).

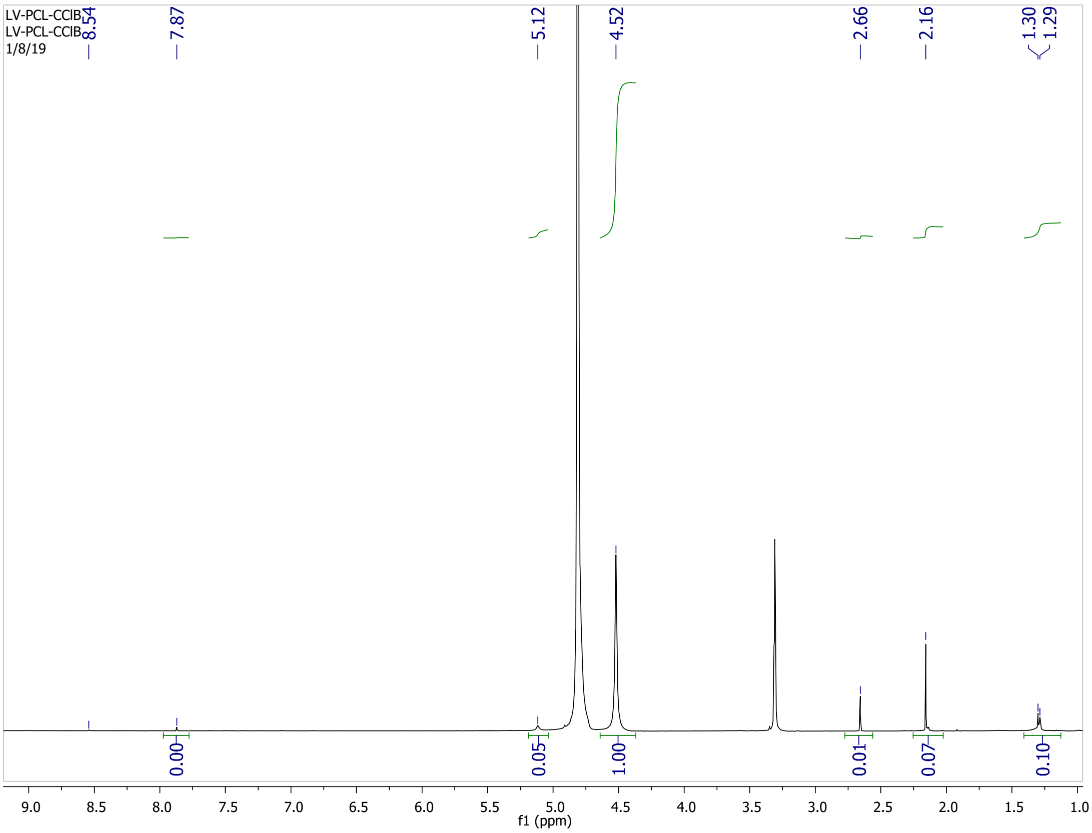

Figure S4. ^{1}H NMR spectra of curcumin control in CD_3OD (*residual solvent signals).

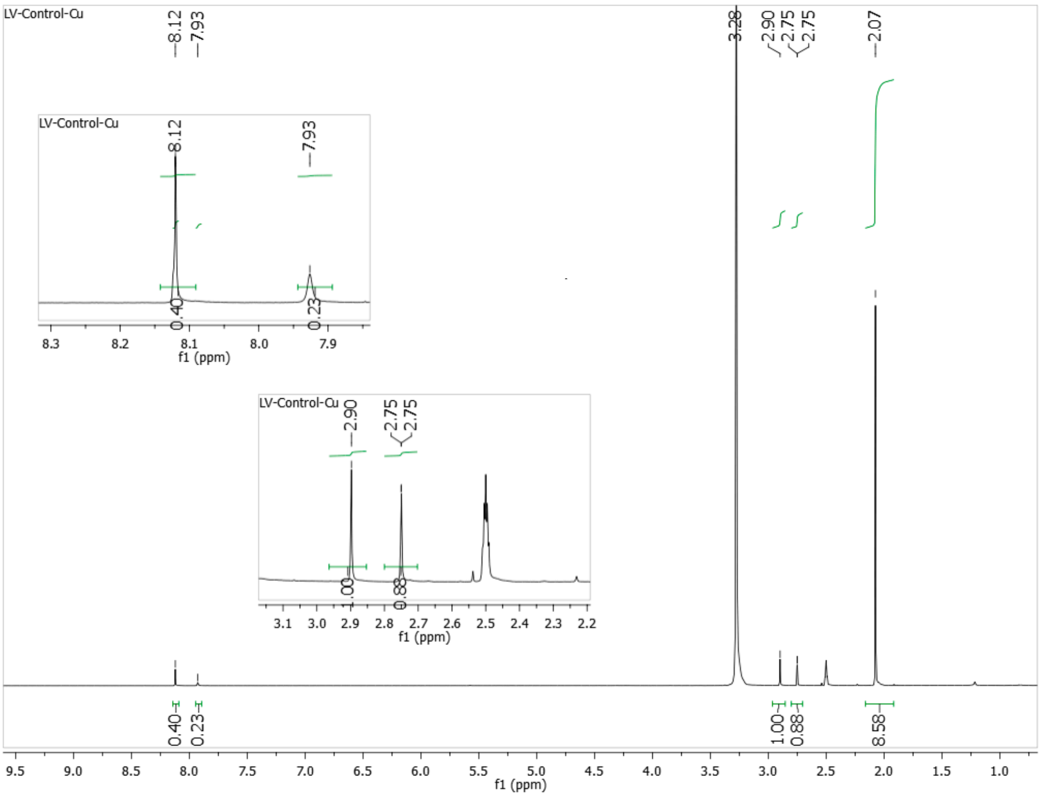

Figure S5. ^{1}H NMR spectra of curcumin control in $DMSO_6$ (*Residual solvent signals).

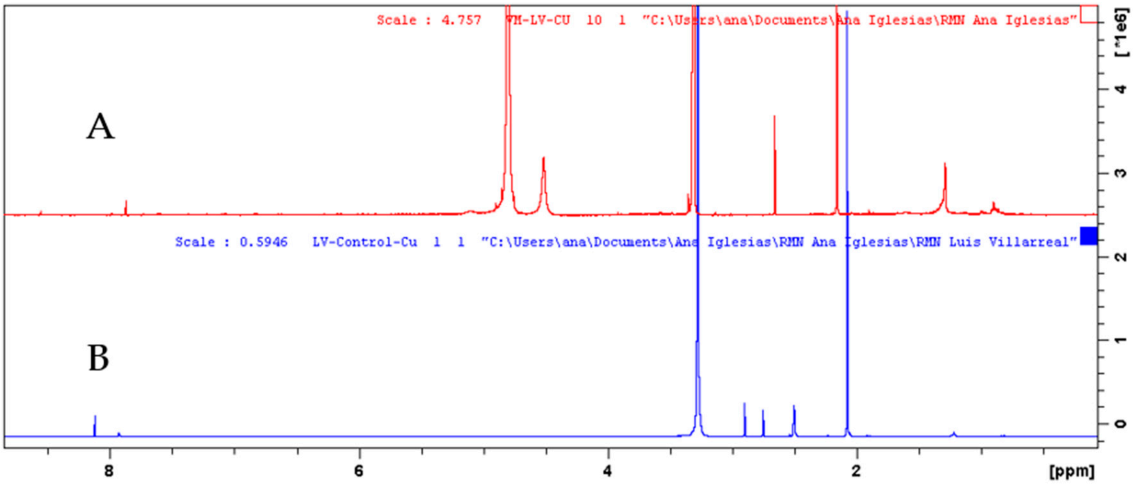

Figure S6. Comparison between curcumin leaves in CD_3OD (**A**) and $DMSOd_6$ (**B**). (* Residual signals in deuterated solvents).

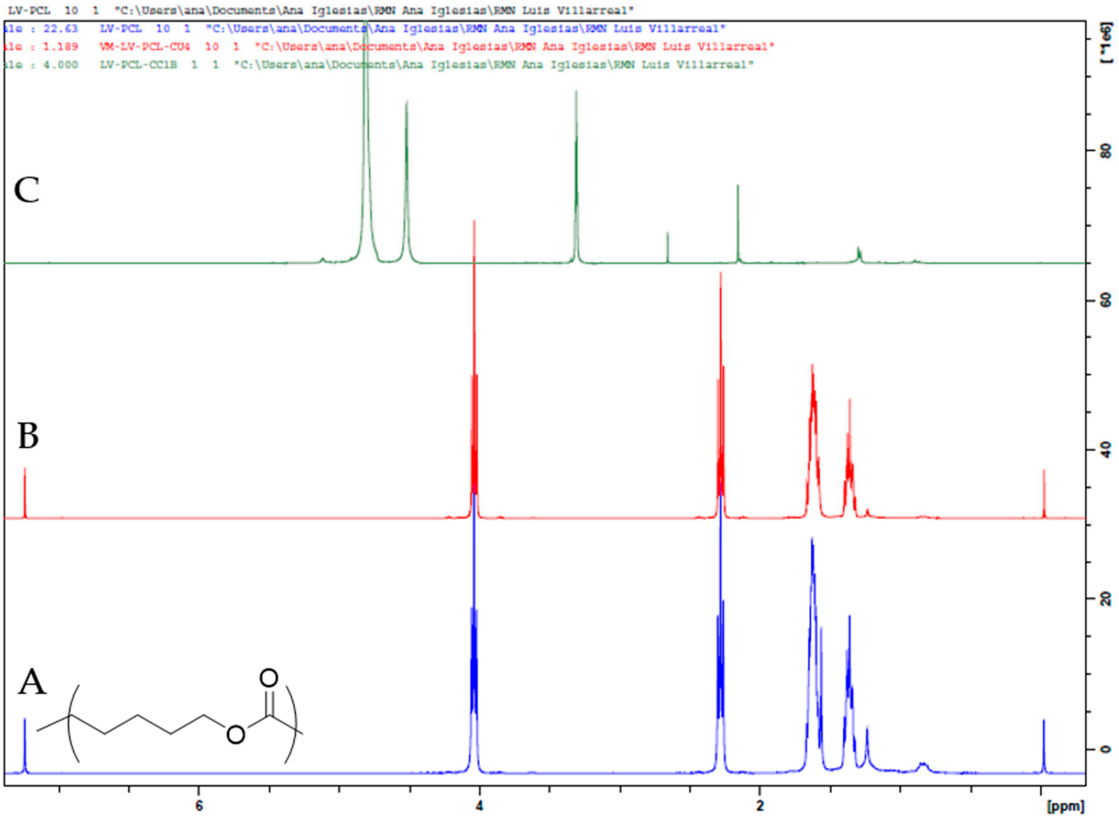

Figure S7. Comparison of ^1H RMN spectra of (**A**) PCL control electrospun fibers in $CDCl_3$, (**B**) PCL-CUR4 fibers in $CDCl_3$ and (**C**) CUR control (CD_3OD).

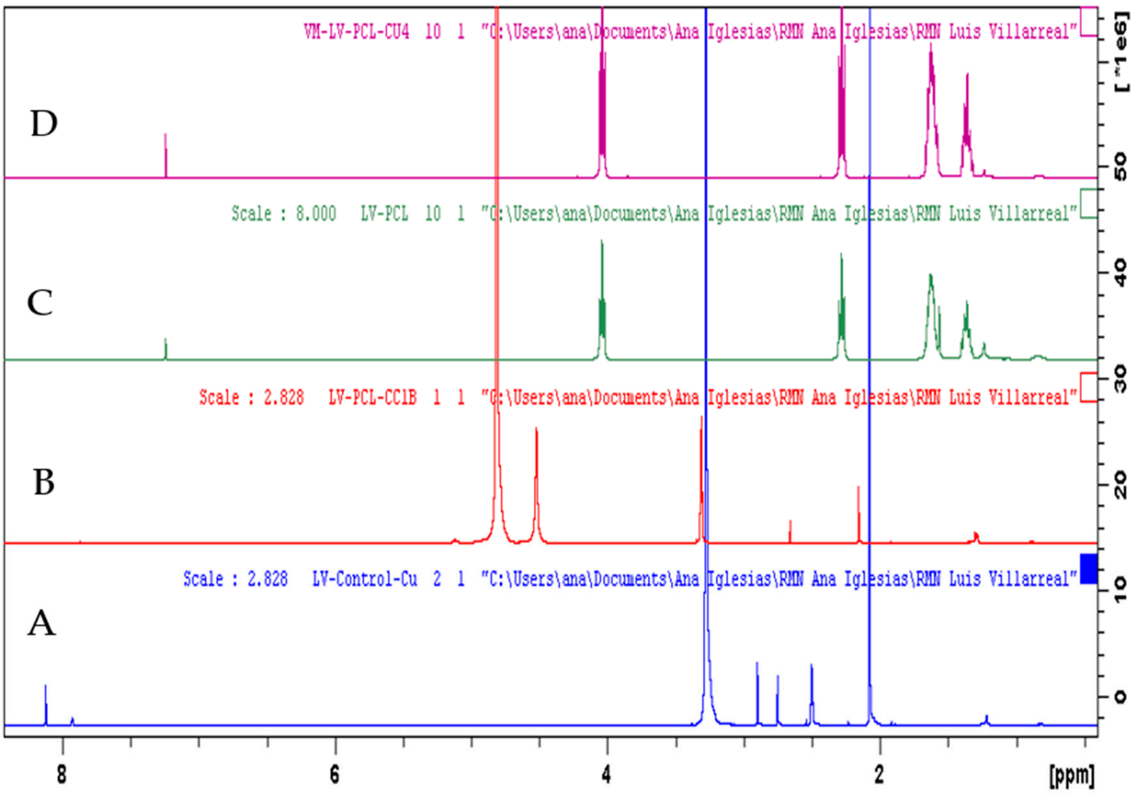

Figure S8. Comparison between (**A**) CUR leaves in $DMSOd_6$, (**B**) CUR leaves in CD_3OD, (**C**) PCL control fibers ($CDCl_3$) and (**D**) PCL-CUR4 fibers ($CDCl_3$), * Residual signals of deuterated solvents.

© 2020 by the authors. Submitted for possible open access publication under the terms and conditions of the Creative Commons Attribution (CC BY) license (http://creativecommons.org/licenses/by/4.0/).
